# IGF2 supports glioblastoma growth and immune evasion through a combination of tumor cell-intrinsic and -extrinsic mechanisms

**DOI:** 10.1093/noajnl/vdaf226

**Published:** 2025-10-15

**Authors:** Kyle M Heemskerk, Samir Assaf, Xiaoguang Hao, Shannon Snelling, Mathieu Meode, Rozina Hassam, Orsolya Cseh, Smriti Kala, James Pemberton, Jennifer A Chan, John Gregory Cairncross, Peter Forsyth, Voon Wee Yong, Reza Mirzaei, Samuel Weiss, Franz J Zemp, Hema Artee Luchman

**Affiliations:** Cumming School of Medicine, University of Calgary, Calgary, Alberta; Arnie Charbonneau Cancer Institute, University of Calgary, Calgary, Alberta; Cumming School of Medicine, University of Calgary, Calgary, Alberta; Arnie Charbonneau Cancer Institute, University of Calgary, Calgary, Alberta; Cumming School of Medicine, University of Calgary, Calgary, Alberta; Arnie Charbonneau Cancer Institute, University of Calgary, Calgary, Alberta; Cumming School of Medicine, University of Calgary, Calgary, Alberta; Arnie Charbonneau Cancer Institute, University of Calgary, Calgary, Alberta; Cumming School of Medicine, University of Calgary, Calgary, Alberta; Arnie Charbonneau Cancer Institute, University of Calgary, Calgary, Alberta; Cumming School of Medicine, University of Calgary, Calgary, Alberta; Arnie Charbonneau Cancer Institute, University of Calgary, Calgary, Alberta; Cumming School of Medicine, University of Calgary, Calgary, Alberta; Standard BioTools, Canada Inc, Markham, Ontario; Standard BioTools, Canada Inc, Markham, Ontario; Cumming School of Medicine, University of Calgary, Calgary, Alberta; Arnie Charbonneau Cancer Institute, University of Calgary, Calgary, Alberta; Cumming School of Medicine, University of Calgary, Calgary, Alberta; Arnie Charbonneau Cancer Institute, University of Calgary, Calgary, Alberta; Mofitt Cancer Center and Research Institute, Tampa, Florida (P.F.); Cumming School of Medicine, University of Calgary, Calgary, Alberta; Department of Oncology, University of Alberta, Edmonton, Alberta; Cross Cancer Institute, University of Alberta, Edmonton, Alberta; Cumming School of Medicine, University of Calgary, Calgary, Alberta; Arnie Charbonneau Cancer Institute, University of Calgary, Calgary, Alberta; Cumming School of Medicine, University of Calgary, Calgary, Alberta; Department of Biochemistry and Molecular Biology, University of Calgary, Calgary, Alberta; Cumming School of Medicine, University of Calgary, Calgary, Alberta; Arnie Charbonneau Cancer Institute, University of Calgary, Calgary, Alberta

**Keywords:** brain tumor stem cells, glioblastoma, insulin-like growth factor 2, multiomics, syngeneic model

## Abstract

**Abstract:**

BackgroundGIntratumoral and intertumoral heterogeneity combined with immunosuppressive tumor microenvironments (TME) contribute to the poor outcomes associated with glioblastoma (GBM). Well-characterized immunocompetent models that recapitulate human GBM features are urgently needed to identify targets in the TME and develop novel therapeutics. Here, we used multiomic approaches to characterize syngeneic mouse brain tumor stem cell lines *in vitro* and in orthotopically engrafted tumors.

**Methods:**

Whole-genome sequencing, transcriptomics, ATAC-sequencing, and imaging mass cytometry were used to characterize syngeneic brain tumor stem cell lines derived from *Trp53^+/−^/Nf1^+/−^* C57Bl6 mice. Mouse and human bulk, single-cell, and spatial sequencing datasets were analyzed for validation. CRISPR/Cas9 and shRNA were used for gene knockdowns. Tumor growth was investigated using orthotopic engraftment in syngeneic C57Bl6 mice.

**Results:**

One of the syngeneic lines, mBT0309, generated tumors with histopathological characteristics of GBM. mBT0309 displayed amplification and high expression of *Igf2*. Copy number gains at the *IGF2* locus were observed in human GBM tumors and stem cell lines. Furthermore, we determined that high *IGF2* RNA expression is associated with poor survival in GBM patients. Imaging mass cytometry on mBT0309 tumors showed early infiltration of monocyte-derived macrophages, vascularization, and cell states characteristic of human GBM. Genetic targeting of *Igf2* decreased *in vitro* cell growth, improved survival of engrafted mice, and decreased the percentage of Arginase-1+ macrophages in mBT0309 tumors.

**Conclusions:**

mBT0309 is a valuable syngeneic model for studying immunosuppression and therapeutic resistance in GBM. IGF2 offers promise as a valuable therapeutic target to combat tumor growth and immunosuppression in GBM patients.

Key Points

- mBT0309 is a preclinically relevant murine syngeneic brain tumor model with *Igf2* amplification.
- *Igf2* contributes to murine GBM development by promoting tumor cell growth and macrophage polarization, correlating with immunosuppression.

Importance of the study

The newly characterized mBT0309 syngeneic mouse model harbors an amplification of *Igf2*, which contributed to its tumor growth *in vivo. IGF2* has been previously found to be upregulated in a subset of GBM tumors. We validated this finding in multiple human GBM tumor datasets and reported copy number gains at the *IGF2* locus in human tumors and GBM stem cell lines. Using human and mouse single-cell/spatial transcriptomics datasets, we observed that IGF2 receptors were expressed by many cell types in the tumor microenvironment. We find that IGF2 promotes an increase in Arginase-1+ macrophage and tumor cell growth in mBT0309, further implicating IGF2 as an important target for precision and immunotherapies for GBM patients.

GBM is a complex disease with extensive cellular and molecular intrapatient and interpatient heterogeneity, with GBM stem cells further contributing to therapeutic resistance and tumor recurrence.^1^^,^^2^ Comprehensive annotation of patient-derived GBM stem cell cultures by our group and others has helped delineate the extensive patient-specific cellular and molecular heterogeneity *in vitro.*^1^^,^^3^ However, there have been few advances in the standard of care in the last 20 years. While immunotherapies have generated promising results in other cancers, their potential as therapeutic options for GBM is still unclear (reviewed in Liu et al^4^). This is partially due to the lack of relevant preclinical immunocompetent models that recapitulate the heterogeneity and complexity of the tumor microenvironment (TME) in human GBM.

To address the increasing need for relevant models, other groups have characterized existing syngeneic brain tumor models and developed new models.^5–7^ Immunological profiling of 4 highly used models showed that certain syngeneic cell lines can generate tumors that closely resemble human GBM tumors.^5^ Likewise, characterization of mouse brain tumor stem cell cultures (mBTSCs) generated from *Qk*/*Trp53*/*Pten* triple-knockouts showed that these cell lines recapitulated immunotherapy responses observed in human patients.^7^ Furthermore, whole-exome analysis of the SB28 and GL261 models revealed that SB28 had a mutational burden that resembled human GBM, and matched checkpoint blockade efficacy observed in human GBM tumors.^6^ While these syngeneic GBM cell lines have provided additional models for preclinical studies, there are still few murine models with multiomic and whole-genome characterization that capture the spectrum of TME heterogeneity.

Our group previously developed a syngeneic murine stem cell model of GBM, mBT0309, from a spontaneous brain tumor in *Trp53^+/-^*/*Nf1^+/-^* C57Bl/6J mice,^8^ which recapitulates the immunoregulatory features of human GBM BTSCs in pre-clinical studies.^9^^,^^10^ Here, we used multiomic approaches, including whole-genome sequencing (WGS), transcriptomic profiling, ATAC-sequencing (ATAC-seq), imaging mass-cytometry (IMC), and *in vitro* and *in vivo* functional studies for in-depth characterization of mBT0309. We further analyzed two additional mBTSC cultures derived from the same colony of *Trp53^+/−^*/*Nf1^+/−^* C57Bl/6J mice to identify specific drivers of tumor growth in these models. We found a focal amplification harbouring several potential oncogenes, including *Igf2* (Insulin-like Growth Factor 2) in mBT0309. IGF2 has been previously shown to play a role in GBM growth^11^ and immunosuppression *in viro*-immunotherapy-treated brain tumors.^12^ We validated high expression of *IGF2* RNA in a subset of GBM tumors in multiple human datasets. We also found that IGF2 receptors are expressed by multiple TME cell types. Genetic targeting of *Igf2* improved the survival of syngeneic engrafted mice and decreased the percentage of Arginase-1+ macrophages. IMC profiling of mBT0309 tumors showed that it recapitulates key features of the human GBM TME. mBT0309 is a valuable tool to add to the suite of syngeneic models, and IGF2 is a promising target in GBM patients.

## Methods

### Derivation of mBTSC Cultures

mBTSCs were derived from C57BL/6J *Trp53^+/−^*/*Nf1^+/−^* mice (gift from Dr Karlyne Reilly) as previously described^8^ and cultured in stem cell-enriched media (EGF (20 ng/mL), bFGF (20 ng/mL), and heparin sulfate (2 µg/mL) (Stem Cell Technologies)^13^ unless otherwise stated. Briefly, C57BL/6J *Trp53^+/−^*/*Nf1^+/−^* mice were monitored for humane endpoint, following which, whole brains were removed and examined for brain lesions, which were microdissected and mechanically dissociated. Murine neural stem cells (NSCs), used as a control for RNA-sequencing (RNA-seq), were isolated from the subventricular zone of wild-type (WT) C57BL/6J mice and cultured under the same conditions.

### Cell Growth and Limiting Dilution Assays

For cell growth analysis, 1000 cells (EGF/FGF condition) or 5000 cells (no growth factor (NOGF) condition) were seeded in a 96-well plate, and cell viability was assessed each day postseeding using alamarBlue (ThermoFisher) as per the manufacturer’s protocol. For limiting dilution assays, 512 cells were seeded in 6 wells of a 96-well plate and serially diluted to 256, 128, 64, 32, 16, 8, 4, 2, and 1 cell per well. Cells were monitored over 1 month for sphere formation and scored as previously described.^14^

### Intracranial Engraftments

mBTSCs (100,000) were implanted into the striatum of the right cerebral hemisphere of C57BL/6J mice (Charles River). Mice were euthanized when the humane endpoint was reached. Brains from each group were formalin-fixed and embedded in paraffin or OCT for sectioning and stained with H&E to assess tumor burden.

### Immunofluorescence

mBTSCs were plated in 8-well chamber slides coated with poly-ornithine and laminin. Cells were fixed with 4% paraformaldehyde and blocked/permeabilized using goat or donkey serum (10%), BSA (2%), and Triton-X100 (0.3%) in phosphate buffer saline (PBS). Cells were incubated with primary antibodies for OLIG2 (ab109186-Abcam-1:100) and SOX2 (ab97959-Abcam-1:500) overnight, then incubated with fluorophore-conjugated secondary antibody (A-11008—Invitrogen) for 1 hour. Cells were counterstained with DAPI and mounted with coverslips. Images were acquired with an Olympus VS110 slide scanning fluorescence microscope or a Leica SP8 confocal microscope. Quantification was performed using QuPath.^15^

### Immunohistochemical Staining

For formalin-fixed paraffin-embedded sections, heat-mediated antigen retrieval was performed with TRIS-EDTA pH 9. For formalin-fixed OCT cryo-sections, heat-mediated antigen retrieval was performed with sodium citrate, pH 6. Sections were blocked and permeabilized using donkey serum (10%), BSA (2%), and Triton-X100 (0.3%) in PBS. Arginase-1 primary antibody (PA5-29645-Invitrogen-1:100) and biotin-donkey-anti-mouse secondary antibody (715-065-151-Jackson) were used with ABC Elite kit (Vector Labs) and diaminobenzidine (DAB) (Sigma) for staining and hematoxylin for counterstaining. Quantification was performed using QuPath^15^ with a single threshold of 0.2 DAB optical density.

### mBTSC WGS

Genomic DNA was isolated from mBT0309 (passage 3 (p3)), mBT0528 (p4), and mBT1116 (p5) using DNeasy Blood and Tissue Kit (Qiagen). Library prep and sequencing were performed by the Centre for Health Genomics and Informatics (CHGI-UCalgary). Reads were aligned to mm10 using BWA-MEM.^16^ Small nucleotide variants (SNVs) were called using bcftools,^17^ copy number variants (CNVs) with cn. MOPS,^18^ and structural variants (SVs) with Smoove.^19^ SNVs and SVs were annotated using ANNOVAR^20^ and Maftools.^21^ g: Profiler^22^ was used for ontology analysis of variants.

### mBTSC RNA-seq

RNA was isolated using the Amersham RNAspin Mini Kit (Cytiva). Library prep and sequencing were performed by the CHGI-UCalgary. RNA was aligned to mm39 using Rsubread,^23^ and differential expression was performed using DEseq2.^24^ Pathway analysis was performed using the Reactome database.^25^

### mBTSC ATAC-seq

ATAC-seq was performed using a previously published protocol^26^ with one modification during library preparation; Size selection (150-400bp) was performed with AmpureXP beads. Library prep and sequencing were performed by the CHGI-UCalgary. For comparative analysis, a previously published dataset with cultured mouse NSCs^27^ was used, and we downsampled our data to the read depth of that dataset. FASTQs were aligned and processed using the protocol described for WGS. ATAC-seq was analyzed using DiffBind^28^ and HOMER.^29^

### Analysis of Single-Cell and Spatial Transcriptomics

Single-cell and spatial transcriptomic libraries from mBT0309 tumors were developed as described (R. Mirzaei, unpublished data). We reanalyzed and integrated the data with Seurat.V5.^30^ Cell clusters were annotated using Annotation of cell types^31^ and validated using spatial transcriptomic images for anatomical location. CNV inferences were performed as previously described.^2^ For human GBM single-cell RNA-seq, we analyzed expression of specific genes in LeBlanc et al^32^ and Abdelfattah et al.^33^

### Analysis of Human GBM Bulk WGS and RNA-Seq

We analyzed our previously published WGS data^3^ for CNVs at the *IGF2* locus. For bulk transcriptomics, RNA was extracted using the AllPrep DNA/RNA Universal kit (QIAGEN). Library prep and sequencing were performed by the CHGI-UCalgary. Reads were aligned to hg38, and DESeq2^24^ was used for normalization and batch correction. Four samples with log_2_ normalized counts of IGF2 above the 95th percentile were compared to the rest of the cohort for differential expression analysis. The threshold for significance was an absolute log_2_ fold change ≥ 2 and *P* ≤ .05. Deconvolution of bulk RNA-seq from tumors was performed using CIBERSORTx^34^ with a single-cell reference matrix from Varn et al.^35^  *IGF2* expression in Varn et al,^35^ was visualized using cBioPortal.^36^ Survival analysis was performed using the GBM IDH WT TCGA cohort^37^ grouping samples with high *IGF2* RNA expression as the top 33% of tumors and low *IGF2* RNA expression as the bottom 66%.

### CRISPR/Cas9 and shRNA Knockdown of IGF2

CRISPR/Cas9-mediated knockdown cells were generated as previously described.^38^ Briefly, cells were lentiviral transduced with Cas9, selected with blasticidin, transduced with guide RNAs targeting IGF2 or the ROSA safe harbor locus as a cut control, and selected using puromycin. For shRNA-mediated knockdown, cells were transduced with shRNA lentivirus targeting IGF2 and a scramble control (GeneCopoeia) and positive cells were selected with puromycin. Knockdown efficiency was tested using Western blot and ELISA.

### Western Blotting and ELISA

For western blotting, cell pellets were lysed in RIPA buffer with sonication. Blots were probed overnight at 4°C with antibodies specific to mouse IGF2 (R&D Systems—AF792) and β-ACTIN (Sigma-A5316) as a loading control. Blots were washed and incubated with HRP-conjugated secondary antibody and imaged with ECL Select and an Amersham Imager 600 (General Electric). For ELISA, conditioned media were collected from mBTSCs seeded at a 100,000-cell density in a T25 flask and allowed to grow for 7 days in EGF/FGF media. A Quantikine ELISA for mouse IGF2 (R&D Systems—MG200) was used to detect the concentration of IGF2 in the conditioned media as per the manufacturer’s protocol.

### Imaging Mass Cytometry

mBT0309 cells were implanted in 12 C57BL/6J mice as described above, and 3 brains were isolated at 7-, 14-, 21-, and 28-day postimplantation. Brains from each time point were formalin-fixed and paraffin-embedded (FFPE). H&E staining was performed to determine tumor burden and select the ideal sections for IMC staining. Brain sections were stained according to the standard IMC staining protocol for FFPE sections, using the panel outlined in Table S1. IMC data were acquired using the Hyperion XTi Imaging System (Standard Biotools). All imaging acquisitions followed the manufacturer’s protocols. Entire tissue sections were screened using the preview mode of IMC for marker-guided selection of regions of interest (ROIs). Selected ROIs were acquired on the same section using the cell mode of IMC, at a resolution of 1 µm and a frequency of 800 Hz. IMC images were analyzed using a previously published protocol^39^ using the deep learning-based workflow.

### Spectral Flow Cytometry

mBT0309 cells were implanted in 5 C57BL/6J mice, as described above, and tumors were isolated at 3.5 weeks postimplantation. Tumors were dissected from the surrounding brain and dissociated using the Tumor Dissociation Kit, mouse (Miltenyi—130-096-730) on the gentleMACS Octo Dissociator (Miltenyi) as per the manufacturer’s protocol. Tumor cells were frozen in CELLBANKER 2 (amsbio—11914) for later flow cytometry using an antibody panel described in Figure S9. Cells were thawed and 2 million cells were stained with a LIVE/DEAD stain followed by Fc Block. Blocked cells were then stained with an extracellular antibody mix for 20 min, followed by fixation/permeabilization with an intracellular antibody stain. Cells were then resuspended in FACS buffer, and flow cytometry was performed on an Aurora 5L (CyTEK). Flow cytometry analysis was performed on FlowJo v11. Data were compared to previously published CyTOF data on GL261 and SB28.^40^

### Statistical Analysis

Statistical analyses were performed using GraphPad Prism Version 10 or R Version 4.3. Specific tests used are described in the figure captions. Unpaired *t* tests were used for the analysis of 2 groups. For analysis of more than 2 groups, ANOVA or Kruskal-Wallis was used with posthoc multiple comparisons between groups. For growth curves, a Gompertz growth model was fit with a sum-of-squares *F* test for group comparison. A χ^2^ test was used for LDAs. Analysis of human GBM tumor RNA-seq deconvolution was performed using multiple Welch’s *t*-tests with FDR correction. For Kaplan-Meier survival studies, median survival and significance were determined by the log-rank test.

## Results

### Syngeneic Murine Glioma Stem Cell Lines Exhibit Differential Tumor Growth in Vivo

**Figure 1.**
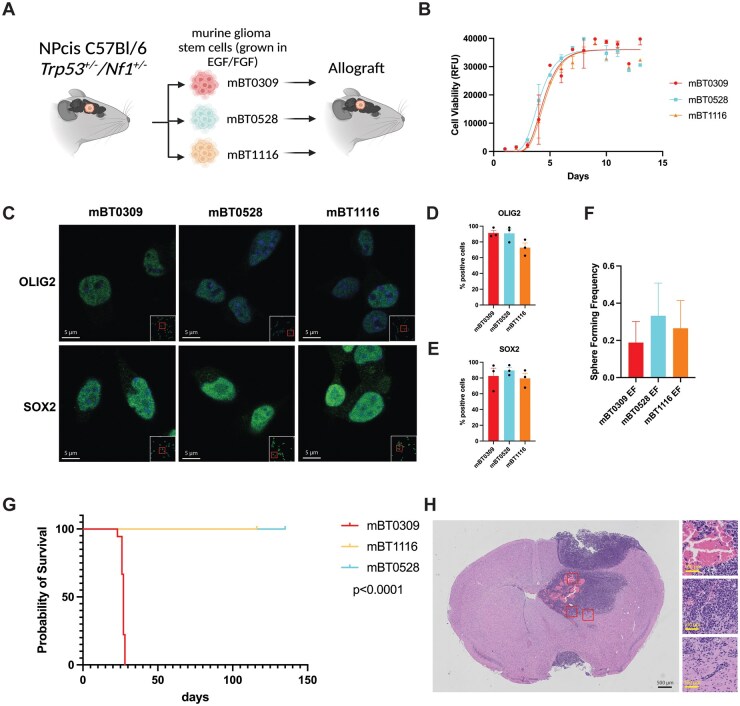
Differential *in vivo* tumor initiation of syngeneic glioma stem cell cultures derived from Trp53+/−/Nf1+/− murine brain tumors. (A) Graphical model of mBTSCs, mBT0309, mBT0528, and mBT1116, their derivation, and *in vivo* growth. Created in BioRender. Heemskerk, K. (2025) https://BioRender.com/kik0nrk. (B) Cell growth over time of the 3 mBTSCs (*n* = 3). Mean +/− SEM. Gompertz growth model line of best fit. (C) Immunofluorescent staining of stem cell markers OLIG2 and SOX2 *in vitro* (representative images from *n* = 3). Scale bars correspond to 5 µm. (D) Percentage of OLIG2-positive cells in the mBTSCs (*n* = 3). Mean +/− SEM. (E) Percentage of SOX2-positive cells in the mBTSCs (*n* = 3). Mean +/− SEM. (F) Sphere forming frequency of the 3 mBTSC lines. Mean +/− max/min estimate (*n* = 6). (G) *In vivo* survival of orthotopically engrafted mBTSC lines in syngeneic hosts. *P* < .0001, Log-rank test (*n* = 10 per mBTSC line, 100,000 cells per mouse). (H) H&E staining of humane endpoint brain tumors in mBT0309 engrafted mice (representative image from *n* = 3). Scale bar corresponds to 500 µm in the large image and 100 µm in the zoom-in images.

We comprehensively characterized 3 mBTSCs, mBT0309, mBT0528, and mBT1116, previously derived from spontaneous brain tumors in *Trp53^+/−^/Nf1^+/−^* C57Bl/6J mice to investigate their preclinical relevance (Figure S1A and B). First, we profiled the growth of all 3 mBTSCs *in vitro* and *in vivo* (Figure 1A). The mBTSCs showed similar growth patterns *in vitro* (Figure 1B) and were all positive for the GBM stem cell markers, SOX2 and OLIG2 (Figure 1C-E). Likewise, all 3 mBTSCs displayed similar sphere-forming frequency (Figure 1F). These results suggest that the 3 mBTSCs maintain the expression of stem cell markers and their self-renewal capacity under stem cell-enriched conditions. Next, we orthotopically engrafted the 3 mBTSC lines in syngeneic mice to determine their tumor initiation potential and found that only mBT0309 generated brain tumors in female mice (Figure 1H and I). As mBT0309 was developed from a male mouse tumor and mBT0528 and mBT1116 were developed from female mice, we further tested mBT0309 for tumor initiation in male mice and found a similar growth pattern (Figure S1C). mBT0309 tumors exhibit histopathological features characteristic of high-grade glioma, including necrosis, microvascular proliferation, and infiltrating tumor cells^41^ (Figure 1I). These findings show that while all 3 mBTSC lines displayed similar self-renewal and growth characteristics *in vitro,* only mBT0309 showed tumor-initiating capacity *in vivo.*

### Whole-Genome Sequencing Reveals Potential Drivers of Tumorigenicity in mBT0309

**Figure 2.**
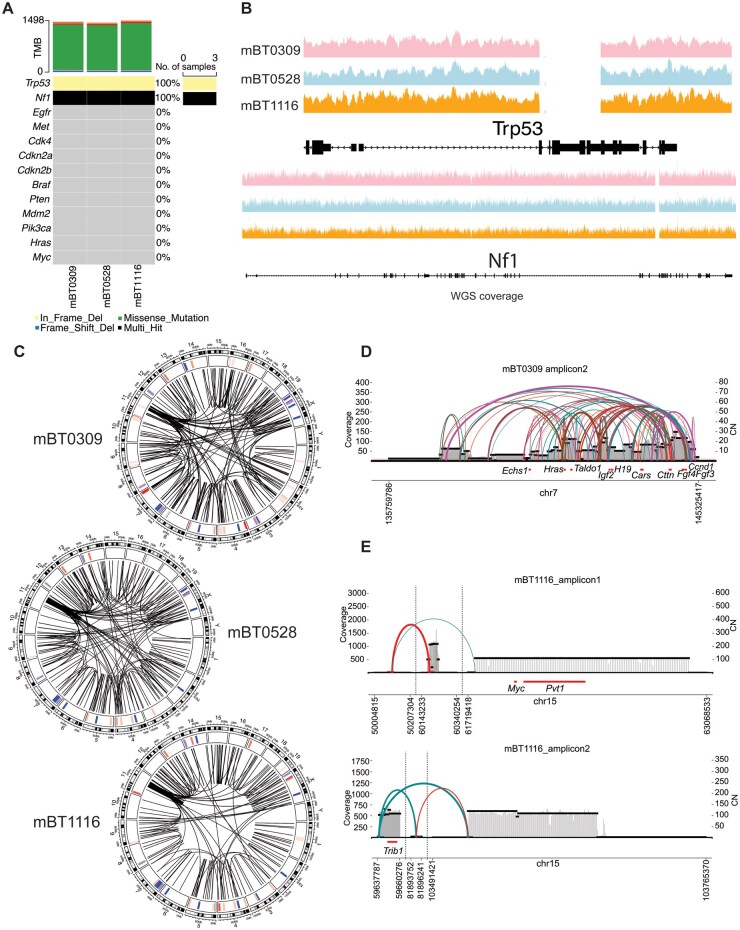
Whole genome sequencing of mBT0309, mBT0528, and mBT1116 reveals differential amplification of oncogenic loci. (A) SNV and SV status of brain tumor-associated genes in mBT0309, mBT0528, and mBT1116. (B) WGS coverage for Trp53 and Nf1 in mBT0309, mBT0528, and mBT1116. (C) SV and CNV landscape of mBT0309, mBT0528, and mBT1116. Chromosomes on the outside track, CNV (Red = Gain, Blue = Loss) status on the middle track, and SVs (intrachromosomal and interchromosomal) in the center. SVs are depicted as lines going from a breakpoint to the other breakpoint. Cell lines compared to mm10. (D) Amplicon prediction for a CNV in Chr7 for mBT0309. (E) Amplicon prediction for two CNVs in mBT1116 on Chr15.

We next asked whether *in vivo* tumor growth could be the result of uniquely acquired mutations in mBT0309. We thus performed WGS on the 3 mBTSC lines and assessed SNVs and SVs. We first examined known brain tumor driver mutations^3^ and found loss of heterozygosity for *Trp53* and *Nf1* in all 3 mBTSC lines (Figure 2A and B). These results indicate that additional mutations are needed to drive tumor growth of engrafted mBTSCs in this model. We next examined the most common mutations present in the 3 mBTSCs, which identified genes not previously reported to be brain tumor drivers (Figure S2A). Overall, the coding mutation burden was similar in all 3 mBTSCs, at approximately 1,450, which is much lower than the commonly used syngeneic glioma model GL261^6^ and closer to that of human BTSCs^3^ (Figure 2A, Figure S2A, and Table S2). GL261 and SB28 are reported to harbor a mutational burden of 4,978 and 108, respectively,^6^ placing these models between the other 2 common models. Gene mutations unique to each of the mBTSCs were limited to 76 in mBT0309, 42 in mBT0528, and 78 in mBT1116 (Figure S2B). Gene ontology analysis of the unique mutated genes in mBT0309 revealed enrichment of genes associated with histone H3K4 methylation (Figure S2C). Coding SNVs in *Setd1a, Setd1b,* and *Kmt2b* were predicted to result in missense mutations, however, the variants identified have not been previously described (Table S3). Gene ontology analysis of mBT0528 showed no term enrichment, while mBT1116 showed enrichment of developmental process genes (Figure S2D). Taken together, these results suggest that mBT0309 tumorigenicity could be driven by unique mutations in epigenetic modifiers.

Next, to fully understand the genetic landscape of the mBTSCs, we performed CNV analysis and overlayed it with the structural variants across the genome (Figure 2C). We observed a highly similar pattern of SVs between the 3 mBTSCs (Figure 2C and Table S3). Conversely, CNV analysis displayed unique regions in all 3 mBTSCs with copy number gains or losses (Figure 2C). We further ran Amplicon Architect^42^ to find potential oncogene amplifications within these unique CNVs. Amplicon Architect identified a large and complex amplification near the end of chromosome 7 (Chr7) in mBT0309 that harbored many breakpoints and fusions, exhibiting a chromothripsis pattern (Figure 2D). We identified potential oncogenes, including *Igf2, Hras, Fgf3, Fgf4,* and *Ccnd1* in this amplicon that could promote tumor growth in mBT0309 (Figure 2D). Similarly, 2 amplicons were identified on chromosome 15 in mBT1116, including the oncogenes *Myc*, *Pvt1* and *Trib1* (Figure 2E). In contrast, no known oncogene amplifications were identified in mBT0528 (Table S4). These findings suggest that the unique amplicon, harboring many potential oncogenes, could be a key genetic alteration contributing to mBT0309 tumor growth.

### Transcriptional and Epigenetic Profiling of mBT0309 Reveals Unique Changes Compared to mBT0528, mBT1116, and WT NSCs

**Figure 3.**
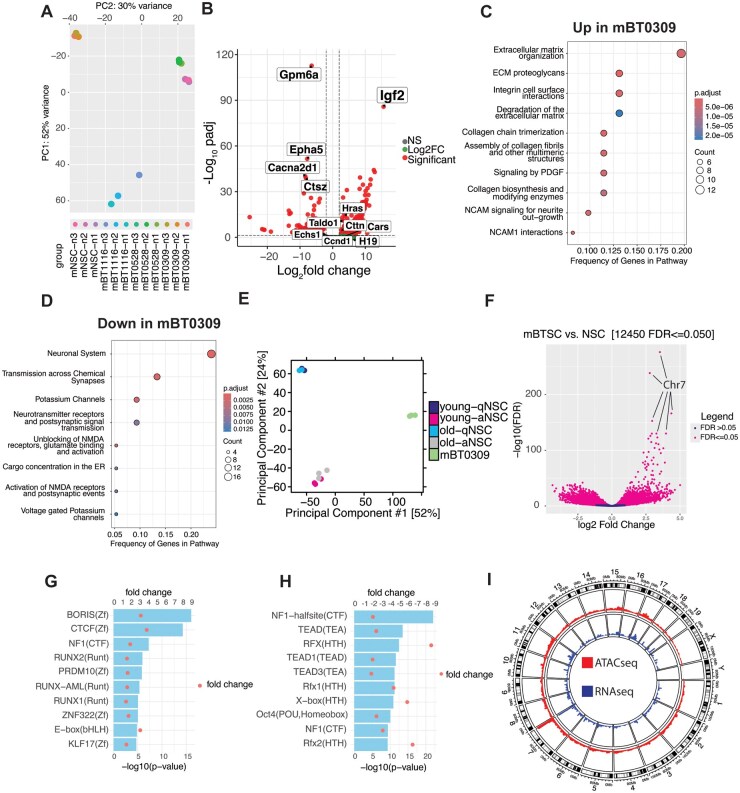
RNA- and ATAC-seq on mBT0309 highlights IGF2 as a potential regulator of tumorigenicity. (A) Principal component analysis of DEseq2 normalized RNA-seq data in the mBT0309, mBT0528, mBT1116, and WT NSCs (*n* = 3). (B) Volcano plot of differentially expressed genes between mBT0309 and mBT0528, mBT1116, and WT NSCs *in vitro*. (fold change ≥ |2|, q-value < .05). (C) Top 10 Reactome pathways enriched in genes upregulated in mBT0309. (D) Reactome pathways enriched in genes downregulated in mBT0309. (E) Principal component analysis of mBT0309 ATAC-seq data compared to WT NSCs cultured *in vitro.* (F) Volcano plot of differentially open chromatin in mBT0309 compared to all populations of NSCs (fold change ≥ |2|, q-value <.05). Fold changes are concentration in mBT0309 minus concentration in all NSC populations (*n* = 3 for mBT0309, aNSCs, and *n* = 2 for qNSCs). (G) Top 10 motifs enriched in ATAC peaks with increased accessibility mBT0309. (H) Top 10 motifs enriched in ATAC peaks with decreased accessibility mBT0309. (I) ATAC-seq and RNA-seq coverage in mBT0309 by genomic location in a circos plot. Representative of *n* = 3.

To further investigate which amplified oncogenes may drive mBT0309 tumor growth compared to the other 2 mBTSCs, we performed RNA-seq on all 3 mBTSCs. We compared the transcriptomic data to WT C57Bl6 NSCs cultured *in vitro.* PCA clustering of the different mBTSCs compared to WT NSCs showed that mBT0528 clustered closest to NSCs, while mBT0309 and mBT1116 clustered on opposite spectrums of PC1 (Figure 3A), perhaps due to differential oncogene amplification. Differential expression analysis showed upregulation of multiple oncogenes on the Chr7 amplicon in mBT0309 compared to mBT0528, mBT1116, and WT NSCs (Figures 2D and 3B, Figure S3A and B, and Table S5). Interestingly, *Igf2* was identified as the most upregulated gene in mBT0309 (Figure 3B). IGF2 has been previously identified as a mediator of GBM cell growth in the absence of EGFR amplification^11^ and linked to immunosuppression *in viro*-immunotherapy-treated GBM and brain metastasis models.^12^ Pathway analysis of differentially expressed genes showed upregulation of extracellular matrix-associated pathways and PDGF signaling (Figure 3C), which could be linked to IGF2 signaling through the PI3K/AKT pathway.^11^ Neuronal system pathways were downregulated (Figure 3D), suggesting a difference in the state of mBT0309 compared to mBT0528, mBT1116, and WT NSCs, which could be partially due to the amplified oncogenes (Figure 2D and E, and Figure S3B).

We also investigated the chromatin landscape in mBT0309, as we observed mutations in histone methylation-associated genes in mBT0309. We performed ATAC-seq on mBT0309 and compared it to previously published ATAC-seq on cultured mouse WT NSCs^27^ (Figure 3E). mBT0309 clustered separately from all populations of cultured NSCs, matching RNA-seq clustering (Figure 3A and E). Differential chromatin accessibility analysis compared to all populations of NSCs identified many differential chromatin accessibility peaks in mBT0309, with the most enriched peaks on Chr7 in the amplicon region (Figure 3F, Figure S3C-E, and Table S6). Motif enrichment analysis of enhanced accessible chromatin peaks in mBT0309 revealed several transcription factors as potential regulators of tumorigenicity, including multiple RUNX transcription factor family members, NFI, and KLF17 (Figure 3G). Interestingly, the top enriched motifs were BORIS and CTCF, which are known to orchestrate 3D chromatin structure to promote treatment resistance in cancer^43^ and IGF2 expression,^44^ respectively (Figure 3G). Motifs enriched in less accessible chromatin peaks in mBT0309 include the developmental transcription factor family RFX, TEAD family members, and OCT4 (Figure 3H). These results suggest that mBT0309 harbors an altered transcriptomic and epigenetic state compared to WT NSCs. To interrogate the relationship between the epigenetic and transcriptomic state, we overlaid the ATAC-seq and RNA-seq coverage (Figure 3I). We observed enhanced gene expression at the Chr7 amplicon, which is consistent with enhanced chromatin accessibility at that location (Figure 3I). This is corroborated by the enrichment of significantly differential accessible peaks on Chr7 in the amplicon region (Figure 3F and Figure S3E). Taken together, these findings suggest that altered chromatin accessibility, particularly at the Chr 7 amplicon, could be responsible for increased expression of oncogenes, including *Igf2,* in mBT0309. *Igf2* was observed to be the most differentially expressed gene in mBT0309; therefore, we sought to determine if *Igf2* could be at the root of the unique *in vivo* tumor growth observed in mBT0309 by investigating its pathway members and receptor expression in single-cell and spatial datasets from mBT0309 and human gliomas.

### Multimodal Analyses of mBT0309 and Human GBM Tumors Suggest That IGF2 Signals to Multiple Cell Types in the TME

**Figure 4.**
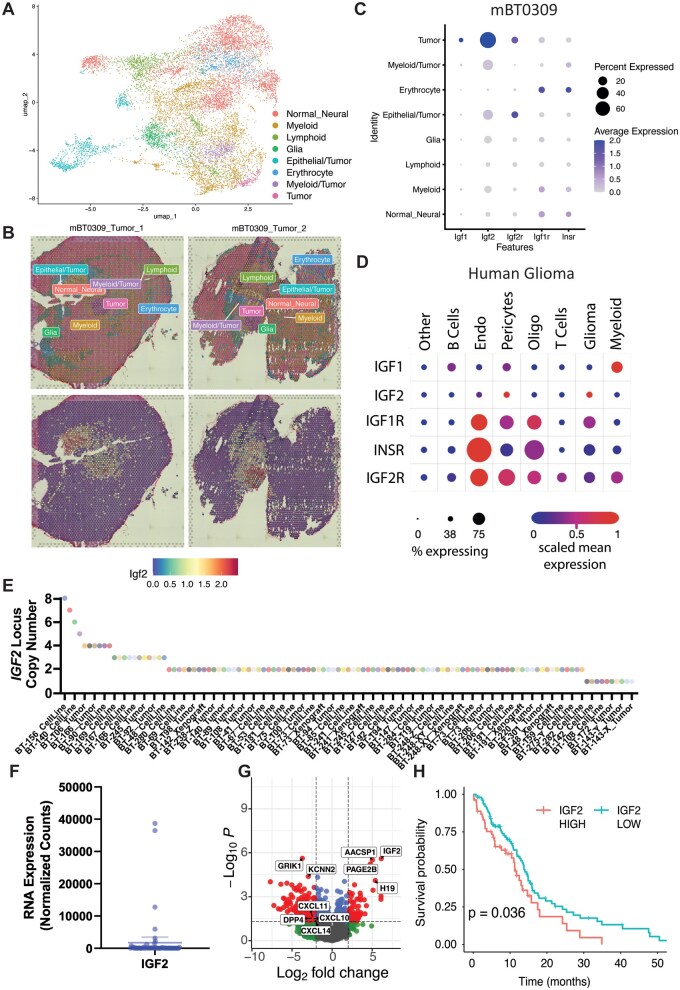
Multimodal transcriptional analysis of IGF2 in mBT0309 and human brain tumors. **(**A) UMAP dimensional reduction plot of mBT0309 tumor single-cell and spatial RNA-seq integration. Clusters of similar transcriptomic programs are denoted by color and annotated using the annotation of cell types. (B) Spatial Dimplot of the cell clusters in two mBT0309 tumors profiled by spatial RNA-seq (upper) and Igf2 expression in those tumors (lower). (C) Dotplot of IGF pathway member expression in the integrated single-cell and spatial RNA-seq from mBT0309 tumors. (D) Dotplot of IGF pathway member expression in single-cell data from human glioma patient tumors. (E) Copy number of the IGF2 locus in human GBM stem cell lines, tumors, and xenografts (*P* < .05, Wilcoxon and Kolmogorov tests). (F) IGF2 RNA expression in GBM tumors (DEseq normalized counts). Mean with 95% confidence interval. *n* = 69 tumors. (G) Volcano plot of differentially expressed genes between IGF2 high (above 95% confidence interval of the mean) and low tumors. (fold change ≥ |2|, *P* < .05). (H) Kaplan-Meier survival curve of human IDH WT GBM patients with IGF2 mRNA high (top 33%) and IGF2 mRNA low (bottom 66%) patients separated. *P* < .036, Log-rank test.

We next queried how IGF2 may promote the growth of mBT0309 *in vivo* using our single-cell and spatial transcriptomic data generated using mBT0309 orthotopic tumors (unpublished data, R. Mirzaei) and validated our results using various human datasets. Integration of single-cell and spatial transcriptomics identified clusters corresponding to normal neural cells, glia, immune cells, stromal, and tumor cells (Figure 4A and B, Figure S4A, and Table S7). Predicted copy number variants in the different clusters validated our findings from WGS with an amplification on Chr7, which was isolated to Tumor, Myeloid/Tumor, and Epithelial/Tumor clusters (Figure 4A and B, Figure S4A). Overall, the composition of mBT0309 tumors resembled the immune profile of other glioma models,^5^ with relatively low numbers of lymphocytes and high myeloid infiltration and diversity (Figure 4A and B). Next, we examined expression of *Igf1* and *Igf2* and their corresponding receptors,^45^  *Igf2r*, *Igf1r,* and *Insr,* in the different cell clusters. *Igf2* was highly expressed in the Tumor cluster, while the other cell clusters exhibited low expression (Figure 4B and C, Table S7). Likewise, *Igf2r* was expressed in Tumor and Epithelial/Tumor cell clusters (Figure 4C, Table S7). Conversely, *Igf1r* and *Insr* were expressed in subsets of all cell clusters, with the highest expression in Normal Neural, Myeloid, and Erythrocyte clusters (Figure 4C). Expression of *Igf1r* and *Insr* in diverse TME cell types suggests that IGF2 could have paracrine signaling effects that create an immunosuppressive tumor-promoting niche. *Igf1* exhibited low expression compared to *Igf2* in the tumor cluster and very low expression in all other clusters, suggesting it had lower importance in this model (Figure 4C). To validate these findings in human tumors, we examined 2 previously published single-cell RNA-seq datasets on Gliomas^33^ and GBM stem cells, explants, and tumors^31^ (Figure 4D and Figure S4B). In the first dataset, *IGF2* was highly expressed in a subset of glioma cells, whereas the *IGF2* receptors were highly expressed in TME cell types, including endothelial cells, pericytes, oligodendrocytes, and myeloid cells (Figure 4D). In the second dataset, all patients had cells expressing *IGF2,* with higher expression in 2 patients (Figure S4C). *IGF2* expression was observed in previously classified cell populations of tumor cells, endothelial cells, and fibroblasts^32^ (Figure S4D). Furthermore, *IGF2* receptors were expressed in subsets of all TME cells, including endothelial, neural, immune cell populations, and tumor cells (Figure S4D and E). *IGF1,* on the other hand, was most expressed in myeloid (Figure 4D) and immune cells (Figure S4E), validating our findings in mBT0309 that glioma cells express IGF2 to a greater degree. These results also suggest that in both mBT0309 and human Gliomas, IGF2 present in the TME could signal to a variety of cell types to promote tumor growth.

To validate our analyses in larger patient cohorts, we next examined our bulk WGS and transcriptomic datasets of GBM tumors and cell lines alongside bulk transcriptomics from external cohorts. In our WGS data for GBM stem cells, tumors, and xenografts,^3^ we found that a subset of patient tumors and GBM stem cells harbored copy number gains at the *IGF2* locus, consistent with our findings in mBT0309 (Figure 4E). In our bulk transcriptomics dataset, 4 patient tumors had IGF2 expression exceeding a 95% confidence interval of the mean (Figure 4F, *n* = 69). We further supported this using the GLASS dataset,^35^ showing ∼10% of patient tumors have high *IGF2* expression, mostly in GBM (Figure S5A and B). These findings confirm that a subset of GBM tumors have high *IGF2* expression. We performed differential expression on the 4 *IGF2* high tumors compared to the rest of our cohort and found higher expression of *IGF2* and *H19*, which are known to be epigenetically co-regulated^44^ (Figure 4G). We found lower expression of *GRIK1* and *KCNN2* in *IGF2* high tumors, which are associated with astrocyte-like glioma cell connectivity^46^ (Figure 4G). Following examination of the list of differential genes, we found that chemokines important for regulating T cell trafficking to tumors, including *CXCL10*, *11*, and *14,* were downregulated in *IGF2* high tumors (Figure 4G and Table S8). Additionally, *DPP4*, a lymphocyte marker,^47^ was also less expressed (Figure 4G and Table S8). Deconvolution of the bulk RNA-seq data revealed that only the T-cell fraction was lower in the *IGF2* high tumors (Figure S5C and D). Moreover, in the TCGA IDH WT GBM patient dataset,^37^ patients within the top 33% of *IGF2* mRNA expression had significantly lower survival (Figure 4H). Therefore, IGF2 is upregulated in a subset of GBM patients and may play a role in immunosuppression to promote disease progression in human GBM.

### IGF2 Promotes Growth under Mitogen-Free Conditions and Tumor Progression in mBT0309

**Figure 5.**
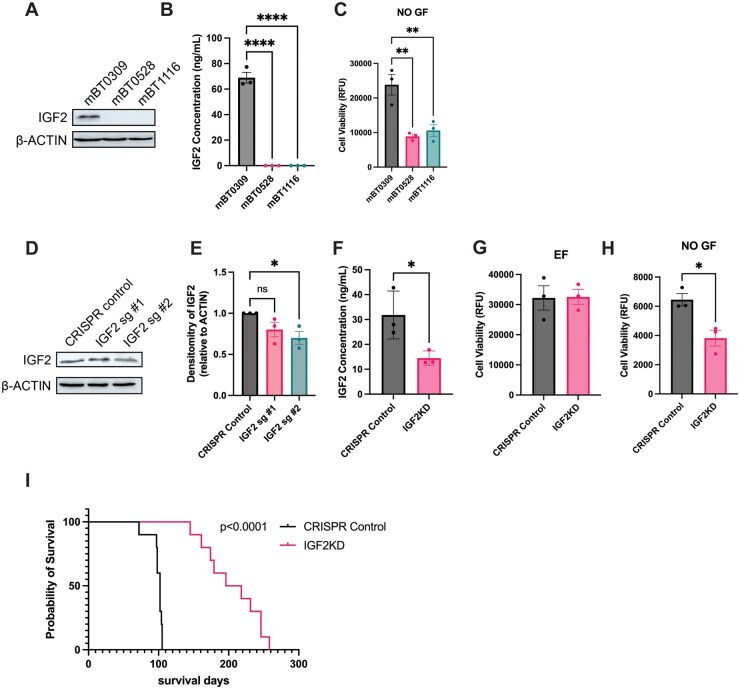
IGF2 supports mBT0309 growth *in vitro* and *in vivo*. **(**A) Western blot of IGF2 in mBT0309, mBT0528, and mBT1116. β-ACTIN as loading control (*n* = 1). (B) IGF2 ELISA on conditioned media isolated from mBT0309, mBT0528, and mBT1116. Mean +/− SEM (*n* = 3). ****; *P* < .0001, ANOVA. (C) Cell viability of mBT0309 compared to mBT0528 and mBT1116 measured at 7 days postseeding in media not supplemented with growth factors EGF and FGF (NO GF) (*n* = 3). **P < .01, ANOVA. (D) Western blot of IGF2 in mBT0309 CRISPR control, IGF2 sg #1, and IGF2 sg #2. β-ACTIN as loading control. Representative of *n* = 3. (E) Densitometry of IGF2 western blots in CRISPR/Cas9 mediated knockdown. IGF2 normalized to β-ACTIN and CRISPR control. Mean +/− SEM (*n* = 3). *;*P* < .05, ANOVA. (F) IGF2 ELISA on conditioned media isolated from mBT0309 CRISPR control and IGF2KD (sg#2). Mean +/− SEM (*n* = 3). *;*P* < .05, t-test. (G) Cell viability of IGF2KD mBT0309 compared to CRISPR control measured at 7 days postseeding supplemented with EGF and FGF (EF) (*n* = 3). (H) Cell viability of IGF2KD mBT0309 compared to CRISPR control measured at 7 days postseeding in NO GF conditions (*n* = 3). *;*P* < .05, t-test. (I) Kaplan-Meier survival curve of CRISPR control (*n* = 10) and IGF2KD (sg#2) (*n* = 10) (100,000 cells per mouse). *P* < .0001, Log-rank test.

Next, we sought to investigate the functional role of IGF2 in mBT0309. First, we examined protein expression of IGF2 in all 3 mBTSCs and found significantly higher IGF2 levels in mBT0309, both in cell lysates and conditioned media, compared to mBT0528 and mBT1116 (Figure 5A and B). To determine if IGF2 could promote mBT0309 growth via autocrine signaling, we cultured the 3 mBTSCs in growth factor-free media (NO GF) (Figure 5C). We observed significantly increased cell viability for mBT0309 compared to mBT0528 and mBT1116, which was not observed in EGF/FGF (EF) supplemented media (Figures 1B and 5C). We next targeted IGF2 using CRISPR/Cas9 in mBT0309, which showed a reduction in the intracellular protein expression but not a full knockout (Figure 5D and E). Likewise, we also observed a 50% reduction in IGF2 levels in conditioned media collected from mBT0309 CRISPR knockdown sg #2 cultures compared to the control (Figure 5F). We also performed shRNA-mediated knockdown of IGF2 in mBT0309, and one shRNA significantly reduced IGF2 protein expression (Figure S6A and B). To determine the effect of IGF2 knockdown on mBT0309 cell growth*,* we cultured the CRISPR controls and IGF2 knockdown in media with EF or NO GF (Figure 5G and H). The IGF2 knockdowns had significantly reduced cell viability after 7 days in the NO GF condition (Figure 5H). We validated this finding in the shRNA IGF2 knockdowns (Figure S6C and D). These data suggest autocrine signaling by IGF2 supports tumor cell growth without supplementation of EGF and FGF, a feature observed in human GBM stem cells.^11^^,^^13^ Next, we tested if IGF2 promotes growth in orthotopic mBT0309 tumors. A significant improvement in mouse survival was observed for IGF2KD mBT0309 compared to control mBT0309 (Figure 5I). We also validated this finding in shRNA IGF2 knockdown mBT0309 (Figure S6E). Taken together, these results suggest that IGF2 supports mBT0309 growth *in vitro* and *in vivo.*

### Spatiotemporal IMC of mBT0309 Growth in Vivo Highlights Vascularization and Myeloid Cell Infiltration in Tumors

**Figure 6.**
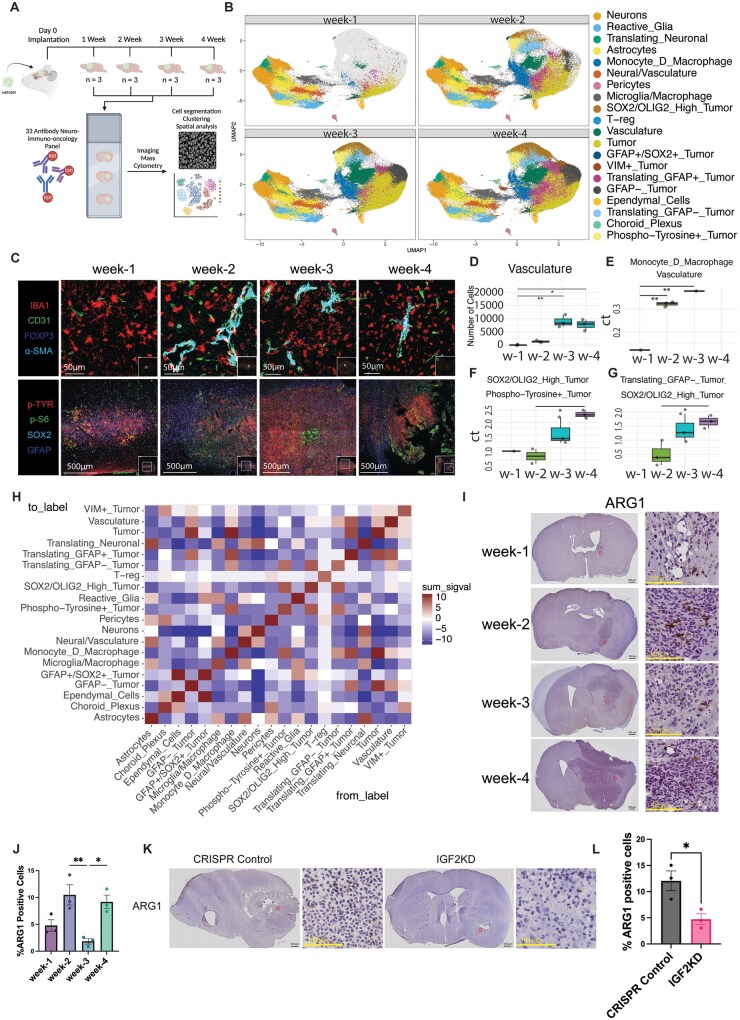
Spatiotemporal profiling of mBT0309 tumors reveals the dynamics of tumor progression. **(**A) A schematic of the imaging mass cytometry (IMC) based profiling of mBT0309 tumor growth. Created in BioRender. Heemskerk, K. (2025) https://BioRender.com/y8mq39f. (B) UMAP of the different cell clusters based on marker expression split over timepoints. (C) Expression of defined markers in mBT0309 tumors over the timepoints. IMC images representative of *n* = 3 for each timepoint. Scale bars correspond to 50 µm in the upper images and 500 µm in the lower images. (D) Quantification of the vasculature cell cluster at the different timepoints (*n* = 3 per timepoint). * and **; *P* < .05 and 0.01, respectively, Kruskal-Walliswith Dunn posthoc multiple comparison. W-# denotes week postengraftment. (E) Interaction of monocyte-derived macrophages (Monocyte_D_Macrophage) with Vasculature cells (Vasculature) in the different timepoints (*n* = 3 per timepoint). No values indicate no significant interaction in that image for that time point. **; *P* < .01, ANOVA with Tukey posthoc multiple comparison. W-# denotes week postengraftment. (F) Interaction of Phospho-Tyrosine+ Tumor with SOX2/OLIG2 High Tumor cells in the different timepoints (*n* = 3 per timepoint). No values indicate no significant interaction in that image for that time point. *; *P* < .05, ANOVA with Tukey posthoc multiple comparison. W-# denotes week postengraftment. (G) Interaction of Translating GFAP- Tumor with SOX2/OLIG2 High Tumor cells in the different timepoints (*n* = 3 per timepoint). No value indicates no significant interaction in that image for that time point. *; *P* < .05, ANOVA with Tukey’s posthoc multiple comparison. W-# denotes week postengraftment. (H) Cell cluster interaction heatmap of all the timepoints combined (*n* = 3 per timepoint). (I) Arginase-1 (ARG1) staining in the different timepoint tumors (Representative of *n* = 3 per timepoint). Scale bars correspond to 500 µm in left images and 100 µm in right images. (J) Quantification of ARG1 positive cells in the different timepoint tumors (*n* = 3 per timepoint). * and **; *P* < .05 and .01, respectively, ANOVA with Tukey’s posthoc multiple comparison. (K) Arginase-1 (ARG1) staining in the CRISPR Control versus IGF2KD mBT0309 tumors from 92 days postengraftment (Representative of *n* = 3 per group). Scale bars correspond to 500 µm in left images and 100 µm in right images. (L) Quantification of ARG1 positive cells in CRISPR Control versus IGF2KD mBT0309 tumors 92 days postengraftment (*n* = 3). *; *P* < .05, *t*-test.

To further understand how the *Igf2* amplified glioma model, mBT0309, shapes the TME over time*,* we performed multiplex IMC using a custom 33-antibody neuro-immuno-oncology panel on a time course of tumor development (Figure 6A, Figure S7A). Unsupervised clustering on segmented cells (Figure S7B) identified 20 different clusters with marker expression of known neural and immune lineages and different populations of tumor cells (Figure S7C and D). Significant changes were observed through tumor development in most cell clusters following quantification, with decreases in neural cell populations and increases in numbers and diversity of tumor cell populations over time (Figure 6B and Figure S8A). Vasculature, monocyte-derived macrophages, and microglia/macrophages also increased throughout tumor development (Figure 6C and D, and Figure S8A). An increase in T-regs was observed at 4 weeks postengraftment, although this was over-represented in 1 brain (Figure 6C and Figure S8A). These results suggest high vascularization and infiltration of myeloid populations and low infiltration of T-cells over time (Figure 6C, Figures S7C and S8A). These results closely resemble other murine glioma models with high myeloid infiltration^5^ and may be influenced by IGF2 paracrine signaling via receptor expression on those cell types (Figure 4C and D). To validate the composition of cells in the mBT0309 tumors, we performed spectral flow cytometry using a custom immune cell panel on tumors isolated at 3.5 weeks postengraftment (Figure S9A-C). We compared mBT0309 spectral flow to CyTOF data from GL261 and SB28 tumors.^40^ The overall proportion of immune cells (CD45+) was much lower in GL261 and mBT0309 compared to SB28, suggesting low overall immune cell infiltration, which is consistent with our IMC data (Figure S9C). The proportion of myeloid and T cells in the CD45+ populations was similar to GL261, with approximately 60% myeloid and 25% T cells, while Dendritic Cells and NK cells had very low representation across the 3 models (Figure S9C). Phenotypic analysis showed that most myeloid cells in mBT0309 are MHC II high activated populations, which is consistent with other models^5^ (Figure S9B and C). T cell phenotyping revealed that mBT0309 had a high ratio of CD4+ to CD8+, a large proportion of which are FoxP3+ T regulatory cells (CD4+) and CD38+ and/or PD-1+ antigen-experienced/suppressed CD8+ T cells, which is also observed in SB28 and GL261 (Figure S9B). Taken together, these results validate the low overall immune cell infiltration into mBT0309 tumors observed by IMC, the overwhelming majority of which are myeloid cells.

Next, using the IMC data, we examined cell-cell interactions to determine if different cell clusters change interactions over time. Monocyte-derived macrophages exhibited early interaction with vasculature, followed by a decrease at later timepoints (Figure 6C and E, Figure S8B). Monocyte-derived macrophages also interacted with multiple tumor cell types early at week 2 but decreased at later timepoints (Figure S8B). We observed a significant increase over time in the interactions between SOX2/OLIG2 high tumor cells and different tumor cell populations, including p-TYR high and translating GFAP- tumor cells (Figure 6C, F, and G, Figure S8B). We also observed a spatial exclusivity for p-TYR and p-S6 expression, which could be indicative of differential cell states in microenvironmental niches (Figure 6C, Figure S7E). Further, each cell type was more likely to interact with itself than with other cell types, and there were multiple interactions between tumor and stromal cells that could influence mBT0309 tumor development (Figure 6H). These data showcase high vascularization, infiltration of monocyte-derived macrophages, and diverse tumor cell state interactions in mBT0309, suggesting that it recapitulates key features of human GBM. IGF2 may regulate these interactions to promote mBT0309 growth.

### IGF2 Promotes Increased Arginase-1-Positive Macrophages

Our analyses of mBT0309 and human datasets suggest that IGF2 may influence tumor growth by signaling to multiple cell types, including myeloid cells and vascular cells. Myeloid cells were found to infiltrate into tumors and interact with vasculature early during mBT0309 tumor development and may be coordinating an immunosuppressive TME that supports mBT0309 growth *in vivo*. These findings support previous reports that IGF2 may polarize macrophages to a more immunosuppressive state.^12^^,^^48^ We did not have an M2-like macrophage marker in our IMC panel; therefore, we further examined macrophage polarization in mBT0309 tumors by staining for Arginase-1 (ARG1) in the time course tumors (Figure 6I). We found a high percentage of ARG1-positive cells in tumors from weeks 2 and 4. These findings suggest polarization at early stages, which may promote tumor growth, and higher numbers at the later stages as a result of tumor inflammation and necrosis (Figure 6I and J and Figure S7A). In IGF2KD tumors, we observed a decrease in the percentage of ARG1+ cells at tumors collected at an early timepoint (Figure 6K and L). At the humane endpoint, there was no significant difference in ARG1+ cells, suggesting that IGF2 may be more important for early polarization (Figure S7F). Taken together, these results suggest IGF2 can promote tumor growth, in part through direct or indirect myeloid cell polarization, which is supported by IGF2 receptor expression in myeloid cells in the TME and high IGF2 expression in a subset of human GBM tumors.

## Discussion

In this study, we used multiomic approaches, including WGS, to fully characterize a syngeneic stem cell model of GBM. We identified an amplification of *Igf2* in mBT0309, which plays a role in tumor growth and immunosuppression. Related to this, we found that there are high levels of myeloid cell infiltration early in mBT0309 tumor development and that IGF2KD led to decreased ARG1+ myeloid cells in the TME. Importantly, using multiple datasets, we validated high *IGF2* expression in a subset of human GBM tumors, which is associated with poor patient survival.

IGF2 was identified early after the discovery of GBM stem cells as a stemness-promoting factor,^11^ but its role in fueling a tumor-promoting microenvironment is only recently becoming apparent. We analyzed mBT0309 and human GBM tumors and found that most microenvironment cells can express IGF2 receptors, suggesting IGF2 could have pleiotropic roles in GBM. Interestingly, a previous study suggested that high-dose IGF2 preferentially activates IGF1R, but low-dose IGF2 activates IGF2R in normal macrophages.^49^ In future studies, it will be important to determine whether IGF2 signaling through its different receptors on TME cell types fuels GBM growth. While IGF1 had low expression in our analysis of single-cell expression data, it is also important to consider the redundancy of these growth factors for potential therapeutic interventions. Nonetheless, it is apparent from this study that IGF2 promotes tumor growth in mBT0309, which could be, in part, due to macrophage polarization. This is consistent with other GBM models^12^ and our analysis of human datasets.

Spatial profiling of human GBM is limited by challenges associated with collecting samples from developing tumors. Using multiplex IMC, we were able to profile the growth of mBT0309 over time in an immunocompetent setting. We found high infiltration of monocyte-derived macrophages, which was associated with vascular interaction during early tumor development. These data were further validated using spectral flow cytometry and were comparable to other murine glioma models.^5^ Spatial profiling of human GBMs has shown interactions between macrophages and vasculature,^50^ which we find are finely tuned temporally. While we did not investigate vascular proliferation in the IGF2KD tumors, receptor expression on vascular cells suggests that there could be a role for IGF2 in vascular function or growth. It will be important to investigate the INS/IGF pathway in all TME cell types in future studies, given its importance in GBM. Interestingly, we further observed spatial exclusivity of p-S6 and p-TYR expression. Hypoxia and HIF-1α are known to be linked to mTOR signaling,^51^ so this observed signaling dichotomy may be coordinated by hypoxia and is indicative of multiple tumor cell states. We highlight the spatial evolution of this GBM model, further confirming the need to target diverse cellular states during tumor evolution.

There are a few murine syngeneic GBM stem cell models that have been previously described,^5–7^ and none that have been fully characterized with the multiomic platforms used in this study. We identified a complex amplification and chromothripsis in mBT0309 that led to high expression of IGF2. Based on the Amplicon Architect output, there are numerous extrachromosomal circular DNAs that could be prevalent in mBT0309. Thus, mBT0309 could also be a useful model to examine the interplay between immunosuppression, stemness, and extrachromosomal DNA. In summary, we characterized mBT0309, a murine syngeneic brain tumor stem cell model that recapitulates many of the features of human GBM and has high relevance for preclinical studies. We identified an amplification of IGF2 and showed that it promotes tumor growth via tumor cell intrinsic and extrinsic mechanisms. IGF2 has potential as a target for GBM treatment and as a biomarker for immune and precision therapy response.

## Supplementary Material

vdaf226_Supplementary_Data

## Data Availability

New sequencing data generated for this manuscript have been deposited in SRA (PRJNA1292979). Our WGS and RNA-seq for human GBM tumors are available at the European Genome-phenome Archive (EGAS00001002709). The GLASS dataset is available at https://www.synapse.org/Synapse: syn17038081. TCGA data are available at https://portal.gdc.cancer.gov/projects/TCGA-GBM. Single-cell RNA-seq used in the study can be found at https://singlecell.broadinstitute.org/single_cell/study/SCP1985/ and GEO: GSE173280. All other data and code used for analysis are available upon request.
