# Supplementary material for: IGF2 supports glioblastoma growth and immune evasion through a combination of tumor cell-intrinsic and -extrinsic mechanisms
Source: Neurooncol Adv. 2025 Oct 15;8(1):vdaf226. doi: 10.1093/noajnl/vdaf226 (PMC12817070; doi:10.1093/noajnl/vdaf226)
Supplement: vdaf226_Supplementary_Data [file vdaf226_supplementary_data.zip › Heemskerketal_Supplemental_figures_revised_Sep_24_2025.docx]

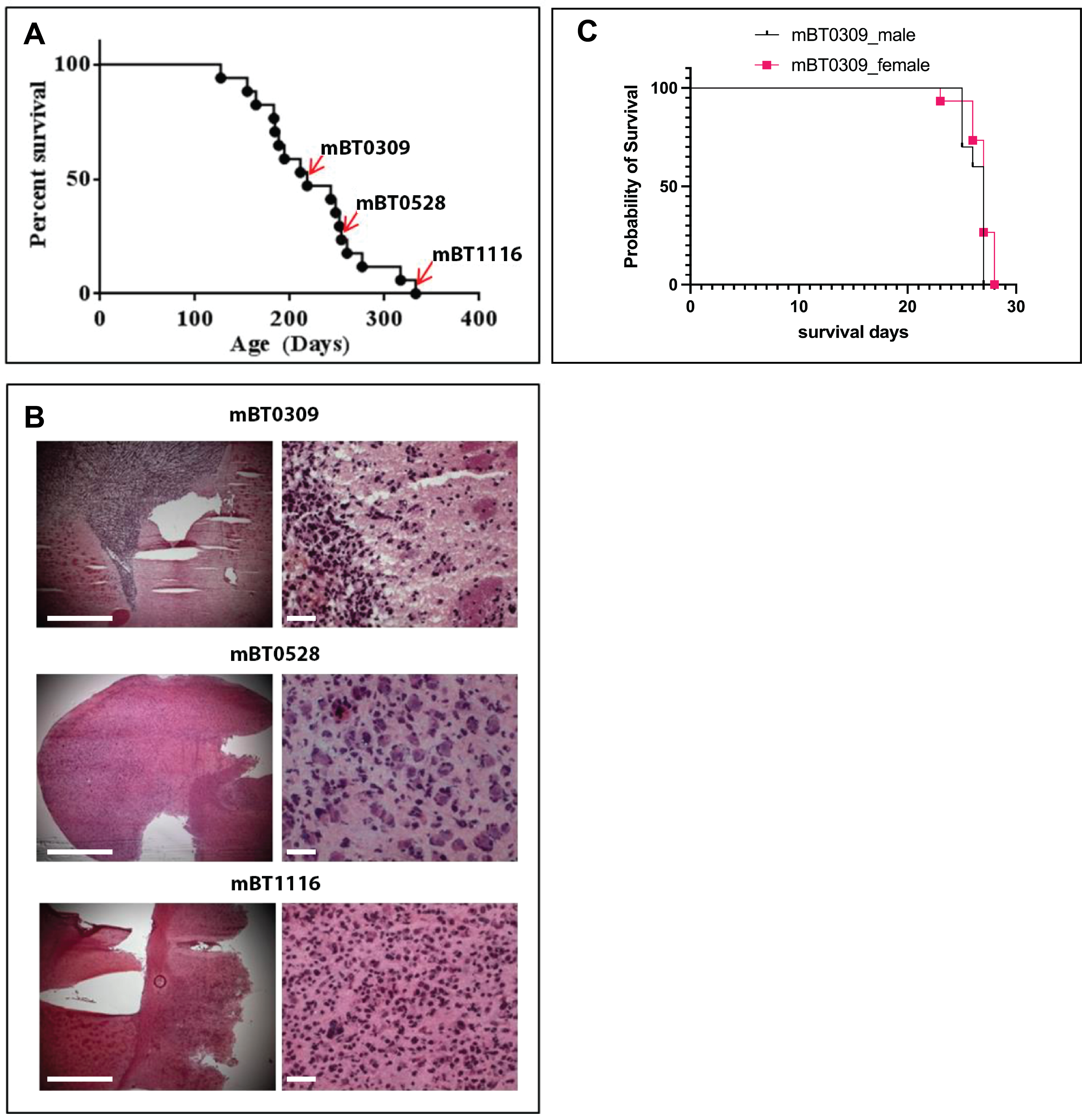


Supplemental Figure S1. **Differential *in vivo* tumor initiation of syngeneic glioma stem cell cultures derived from *Trp53^+/-^/Nf1^+/-^* murine brain tumors.** A) Survival curve of *Trp53^+/-^/Nf1^+/-^* mice with red arrows denoting the animals with spontaneous brain tumors that resulted in cultured mBTSC lines^8^. B) H&E of tumor sections from the brain lesions for each derived mBTSC. Scale bars in images correspond to 2 mm (left) and 200 μm (right). C) Survival curve of male syngeneic mice orthotopically engrafted with mBT0309.


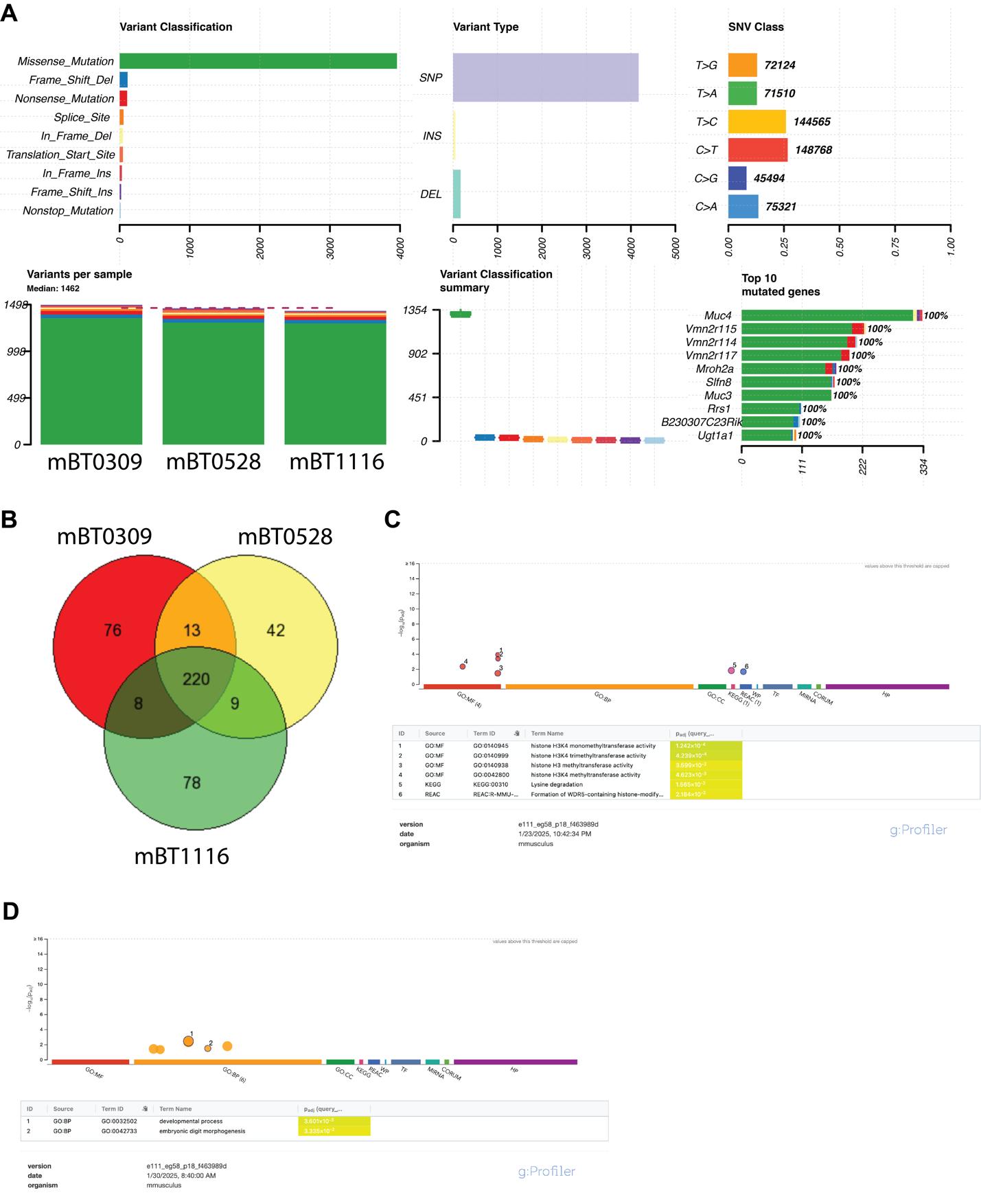


Supplemental Figure S2. **Whole genome sequencing of mBT0309, mBT0528, and mBT1116 reveals differential amplification of oncogenic loci.** A) Summary of the SNV and SV landscape for the three mBTSCs. B) Common and unique genes with SNVs and SVs in the three mBTSCs. C) Gene ontology analysis of genes with SNVs and SVs unique to mBT0309. D) Gene ontology analysis of genes with SNVs and SVs unique to mBT1116.


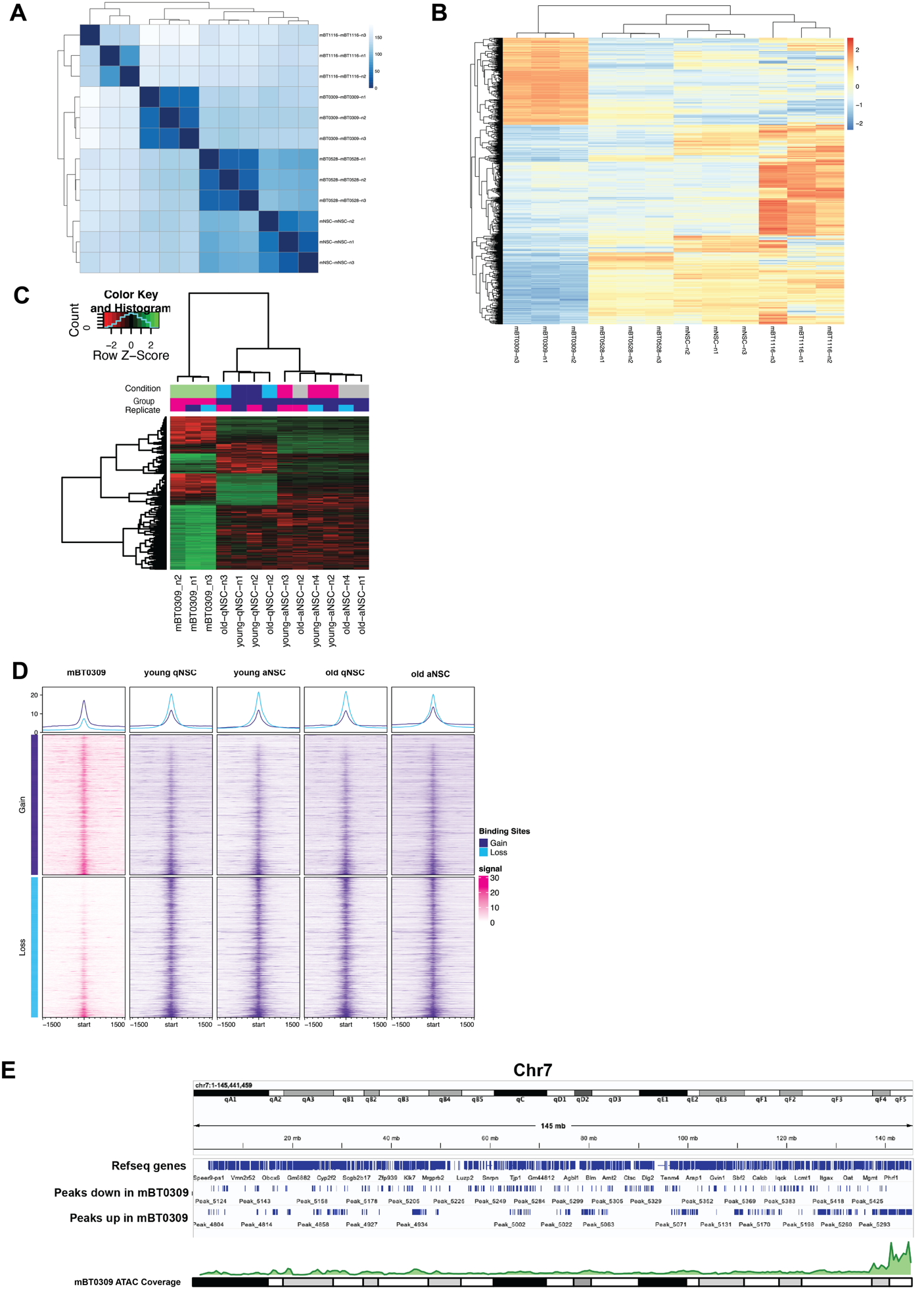


Supplemental Figure S3. **Transcriptomic and epigenetic analysis of mBT0309 compared to mBT0528, mBT1116, and WT NSCs.** A) Sample distances based on DEseq2 normalized RNA sequencing data in mBT0309, mBT0528, mBT1116, and WT NSCs (n=3). B) Heatmap of significantly differentially expressed genes between mBT0309 and all other samples with hierarchical clustering (fold change ≥ |2|, q-value <0.05). C) Heatmap of differentially accessible chromatin regions in mBT0309 compared to WT NSCs with hierarchical clustering. D) ATAC-seq peak profiles of differentially accessible regions between mBT0309 and WT NSCs. E) IGV snapshot of differentially accessible chromatin peaks on Chr7 overlayed with ATAC-seq coverage.


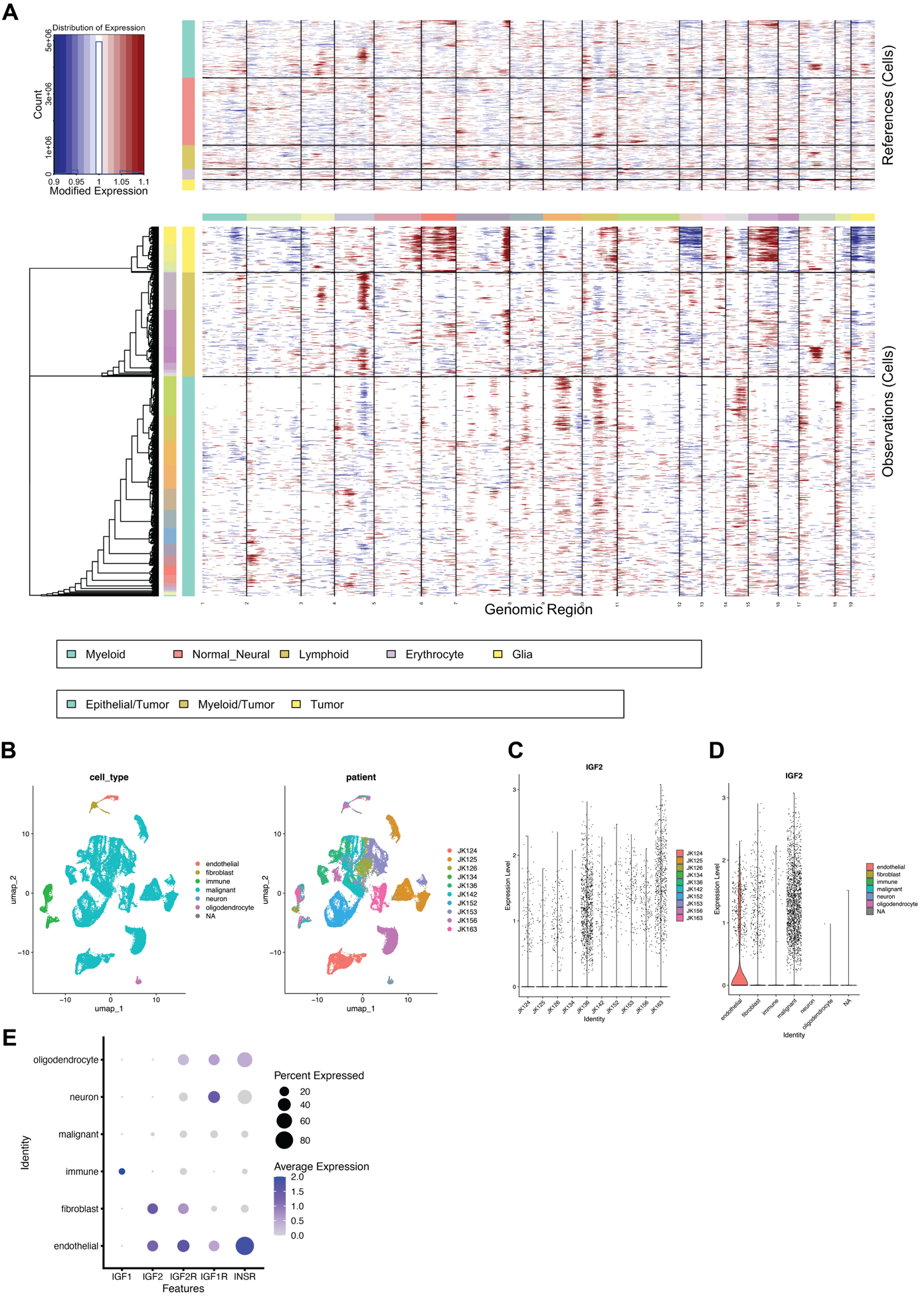


Supplemental Figure S4. **mBT0309 tumor single-cell and spatial RNA-seq, InferCNV, and human single-cell RNA-seq analysis.** A) InferCNV results from mBT0309 integrated single-cell and spatial RNA-seq. B) Dimensional reduction plots from human GBM single cell RNA-seq data^32^. Left is the classification based on cell type, and right is the classification based on patient ID. C) *IGF2* RNA expression in single cells from different patients. D) *IGF2* RNA expression in single cells classified into cell types. E) Dot plot of *IGF1,* *IGF2*, *IGF2R*, *IGF1R*, and *INSR* RNA expression in the cell type clusters.


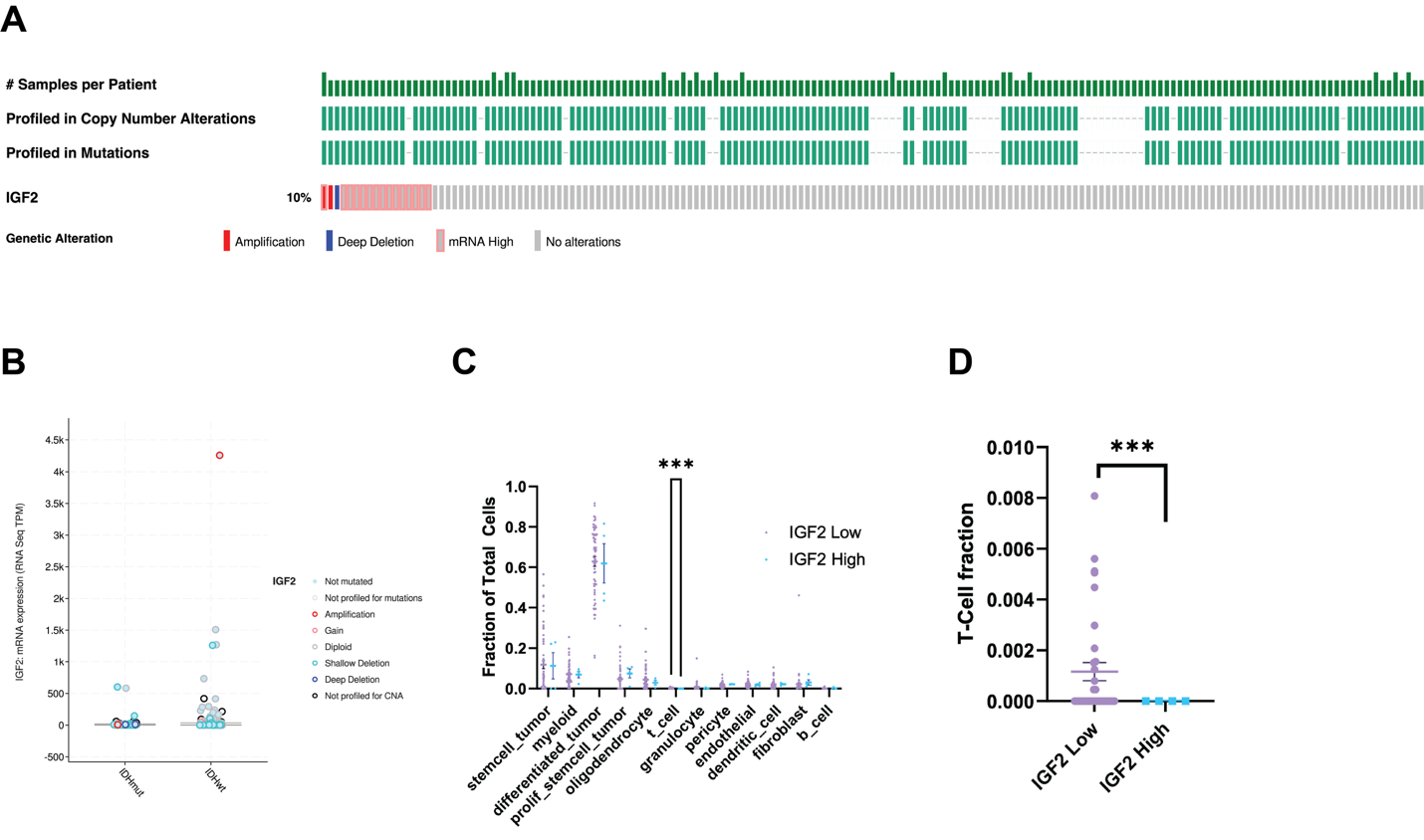


Supplemental Figure S5. **Analysis of IGF2 expression in human bulk RNA-seq datasets.** A) Oncoplot of *IGF2* in the GLASS dataset. B) I*GF2* mRNA expression in IDH mutant (mut) versus IDH wild-type (wt) patient tumours in the GLASS^35^ dataset. C) Predicted proportions of different cell types by CIBERSORT in our GBM bulk RNA seq dataset, separated by *IGF2* high (above 95% confidence interval of the mean) and low expression. ***; p-value <0.001, Welch’s t-test. D) The fraction of T-cells predicted from bulk RNAseq in GBM tumors with high IGF2 expression compared to low IGF2 expression. ***; p-value <0.001, Welch’s t-test. (Zoom in on (I))


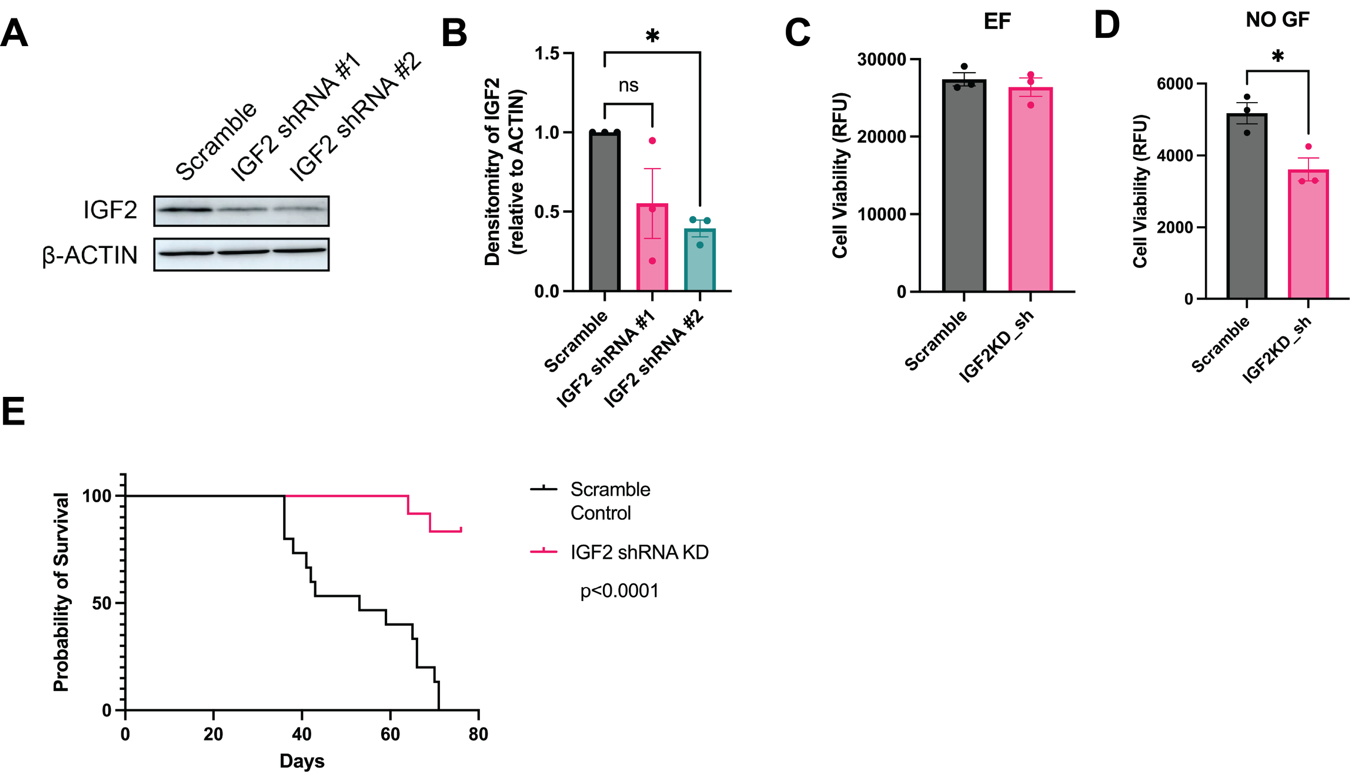


Supplemental Figure S6. **IGF2 shRNA knockdown decreases mBT0309 growth *in vitro* and *in vivo.*** A) Western blot of IGF2 in mBT0309 Scramble control, IGF2 shRNA #1, and IGF2 shRNA #2. β-ACTIN as loading control. Representative of n=3. B) Densitometry of IGF2 western blots in shRNA-mediated knockdown IGF2 normalized to β-ACTIN and Scramble control. Mean +/- SEM (n=3). *; p <0.05, ANOVA. C) Cell viability of IGF2KD (shRNA #2) mBT0309 compared to Scramble control measured at 7 days post-seeding supplemented with EGF/FGF (EF) (n=3). D) Cell viability of IGF2KD (shRNA #2) mBT0309 compared to Scramble control measured at 7 days post-seeding in no growth factor (NO GF) conditions (n=3). *; p <0.05, t-test. E) Kaplan-Meier survival curve of shRNA Scramble control (n=15) and IGF2KD (shRNA #2) engrafted mice (n=12) (100,000 cells per mouse). p<0.0001, Log-rank test.


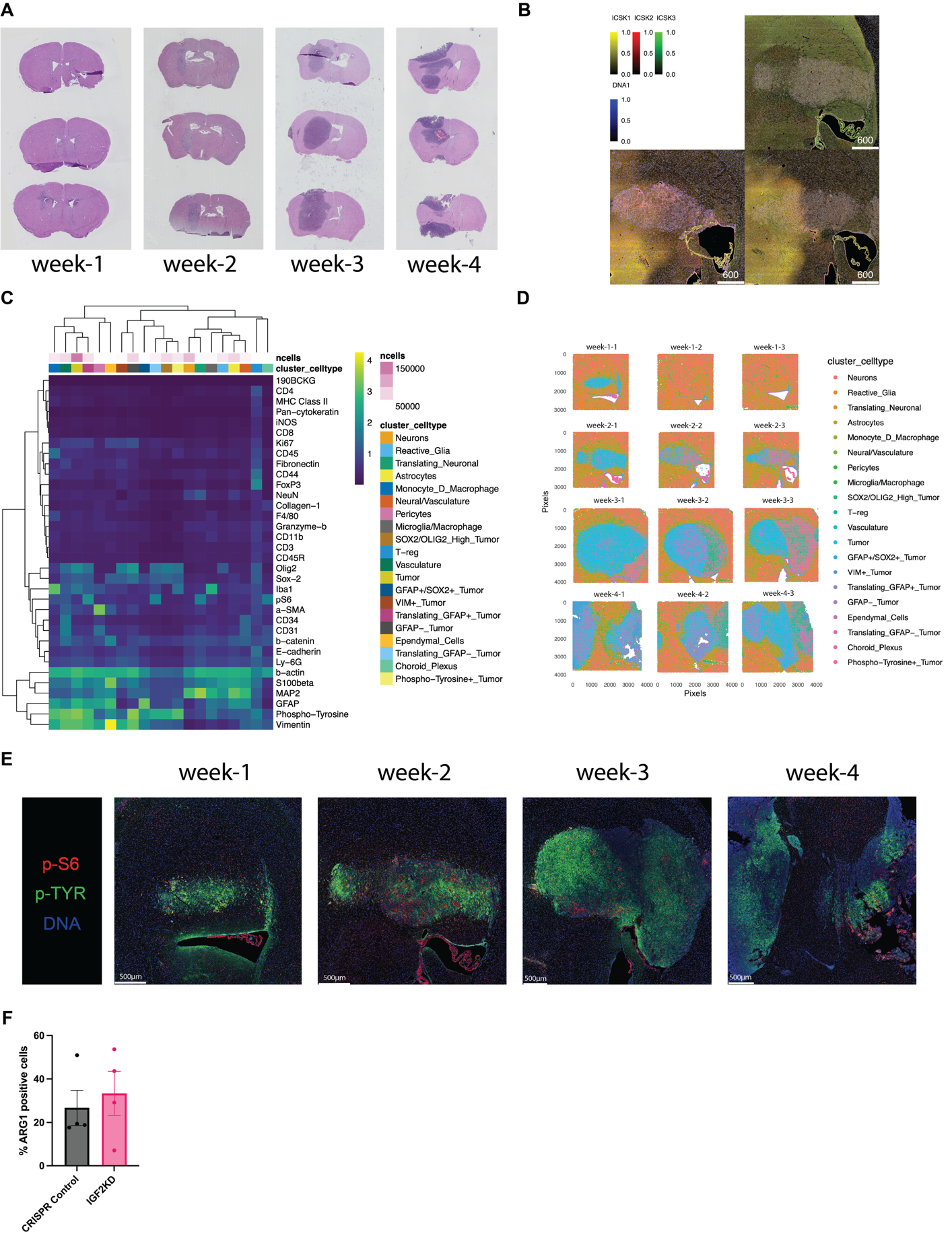


Supplemental Figure S7. **IMC analysis of mBT0309 tumors at different timepoints and ARG1 staining quantification in IGF2KD mBT0309 tumors at endpoint.** A) H&E staining of mBT0309 tumors at 1-, 2-, 3-, and 4-week timepoints post-engraftment (n=3 per timepoint). B) Representative image of cell segmentation in the IMC images using three cytoplasmic markers and two nuclear markers (one DNA shown). Images from week 2. C) Heatmap of averaged marker expression in cells designated to each cell cluster. Hierarchical clustering of cells and markers is depicted on the top and left axes, respectively. D) Spatial location of clustered cell types in each image from the timepoints. W-# denotes week post-engraftment. E) Expression of p-tyrosine (p-TYR) and p-S6 in mBT0309 tumors over the time course. IMC images are representative of n=3 for each week. F) Quantification of ARG1 positive cells in CRISPR Control versus IGF2KD mBT0309 tumors from humane endpoint (n=4). Non-significant, t-test.


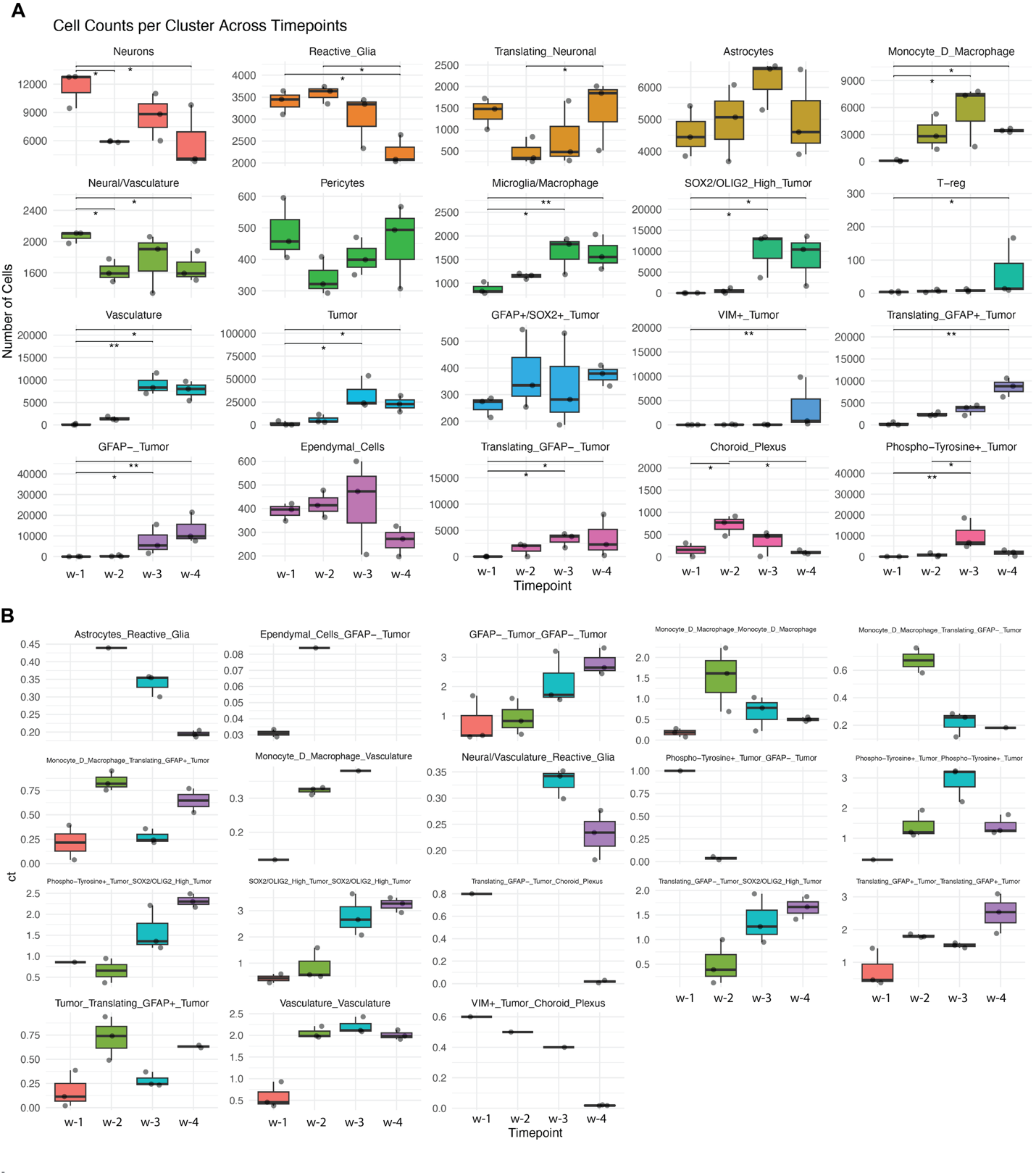


Supplemental Figure S8. **Quantification of cell clusters and statistical analysis of interactions in the IMC mBT0309 tumor time course.** A) Quantification of the different cell cluster cell numbers at the different timepoints. (n=3 per week). * and ** denote p<0.05 and 0.01, respectively, Kruskal-Wallis with Dunn post-hoc multiple comparison. W-# denotes week post-engraftment. B) Quantification of interactions between different cell clusters in the different timepoints (n=3 per week). Only interactions with a significant change over the time course (ANOVA, p<0.05) are shown. No value indicates no significant interaction in that image for that week. W-# denotes week post-engraftment.


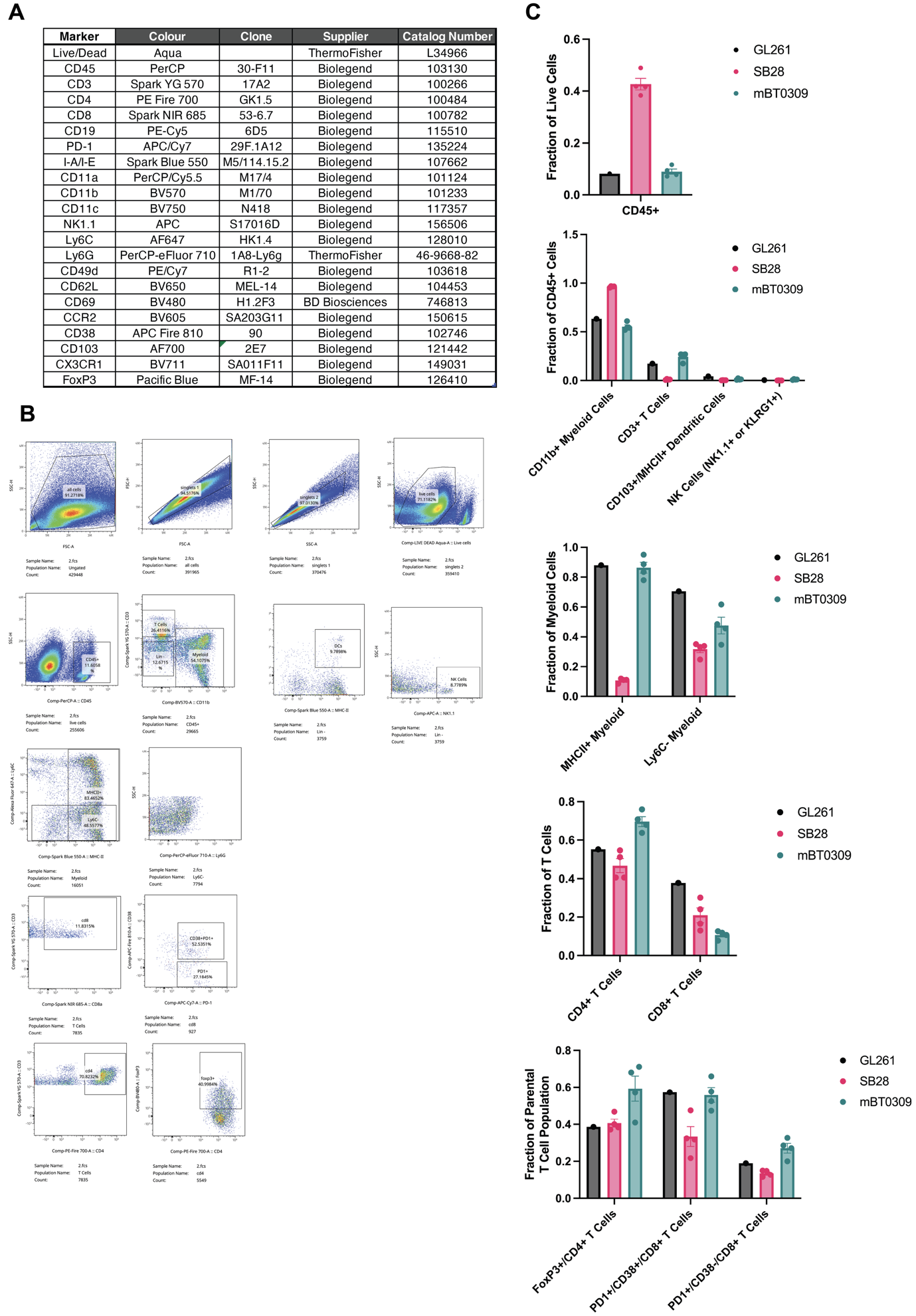


Supplemental Figure S9. **Spectral flow cytometry of immune cell populations in mBT0309 tumors and comparison to GL261 and SB28 CyTOF data**. A) Spectral flow panel used for characterizing mBT0309 tumors. CyTOF data on GL261 and SB28 was previously published, downloaded, and re-analyzed from Simonds *et al.,* 2021^40^. B) Gating strategy for spectral flow analysis of mBT0309 tumors. Gating strategy was replicated for CyTOF data with the same markers, except KLRG1 was used for NK cells. C) Fractions of populations of immune cells in mBT0309 compared to GL261 and SB28 tumors (GL261: n=1, SB28: n=4, mBT0309: n=4). Data represent mean +/- SEM.
